# Identification of Potential Performance-Related Predictors in Young Competitive Athletes

**DOI:** 10.3389/fphys.2019.01394

**Published:** 2019-11-15

**Authors:** Katharina Blume, Bernd Wolfarth

**Affiliations:** ^1^Department of Sports Medicine, Humboldt University of Berlin/Charité University Medicine, Berlin, Germany; ^2^Zentrum für Innere Medizin, Klinikum Garmisch-Partenkirchen, Garmisch-Partenkirchen, Germany; ^3^Institute for Applied Training Science (IAT), Leipzig, Germany

**Keywords:** athlete, competitive sport, intervention, training load, health, stress, performance, immune system

## Abstract

**Introduction:**

Systematic training is an essential demand for the individual success of an athlete. However, similar training modalities cause individual responses, and finally, decide on athletes’ success or failure. To predict performance development, potential influencing parameters should be known. Therefore, the purpose of this study was to identify performance-related parameters in young competitive athletes.

**Methods:**

Individual performance developments of 146 young athletes (m: *n* = 96, f: *n* = 50, age V1: 14.7 ± 1.7 years) of four different sports (soccer: *n* = 45, cycling: *n* = 48, swimming: *n* = 18, cross-country skiing: *n* = 35) were evaluated by analysis of 356 visits in total (exercise intervention periods, 289 ± 112 d). At V1 and V2 several performance parameters were determined. Based on the relative performance progress (Δ), potential influencing predictors were analyzed: training load, health sense, stress level, clinical complaints, hemoglobin, vitamin D, hs-CRP and EBV serostatus. Data were collected within a controlled, prospective study on young athletes, which was conducted between 2010 and 2014.

**Results:**

Athletes improved their performance by 4.7 ± 10.7%. In total, 66.3% of all athletes represented a positive performance progress. This group demonstrated, despite similar training loads (*p* = 0.207), enhanced health senses (*p* = 0.001) and lower stress levels (*p* = 0.002). In contrast, compared to athletes with an impaired performance progress, no differences in hemoglobin values (m: *p* = 0.926, f: *p* = 0.578), vitamin D levels (0.787) and EBV serostatus (*p* = 0.842) were found. Performance progress was dependent on extents of health senses (*p* = 0.040) and stress levels (*p* = 0.045). Furthermore, the combination of declined health senses and rised stress levels was associated with an impaired performance development (*p* = 0.018) and higher prevalences of clinical complaints (*p* < 0.001) above all, in contrast to hs-CRP (*p* = 0.168).

**Discussion:**

Athletes with an improved performance progress reported less pronounced subjective sensations and complaints. In contrast, objective known performance-related indicators, offered no differences. Therefore, subjective self-reported data, reflecting health and stress status, should be additionally considered to regulate training, modify intensities, and finally, predict and ensure an optimal performance advance.

## Introduction

The goal of every competitive athlete is success in his sport. Athletes’ aims are individually different (Bompa, 1995). Senior success requires an optimal performance development over numerous years of systematic training starting at a young age. An uncomplicated passage through this period is indispensable. Therefore, predictors of performance progress and potential risk factors, including their complex interactions, should be known and considered.

Diverse parameters define and characterize an elite athlete: e.g., sport-specific skills, physical performance, anthropometric and physiological characteristics, maturation, genetic predisposition, length of training, experience, health, and psychosocial factors (Armstrong and McManus, 2011; McManus and Armstrong, 2011). In the medical context, health plays an important role. Especially in adolescence, there are known parameters, which can negatively affect the risk of physical and psychological illness and injury: high training loads (Fleisig et al., 2011; Hjelm et al., 2012), an early specialization (Bompa, 1995; Jayanthi et al., 2013), previous illnesses, environmental factors, and negative stressors such as school problems, parental conflicts, pressure to perform, and competition failure (Cohn, 1990; Scanlan et al., 1991).

An improvement of performance can be assessed at different levels: long term until reaching top-level status, short term during training periods for season or competition preparation. Beside sport-specific skills, the development of an adequate endurance capacity is necessary to ensure resilience and to affect health, training and ability for a fast regeneration progress (Borresen and Lambert, 2009; Dhabhar, 2014). For example, road cyclists establish their base for the season in the winter months. This requires a systematic training and the knowledge of strengths, weaknesses and limitations. Inadequate strains, such as to intensive training loads, can lead to a diminished immune competence affecting recurrent infections. Ignoring medical issues can cause frequent interruptions, lack or stagnation of performance, up to retirement from competitive sports (Maffulli et al., 2010).

As part of annual systematic medical examinations, mostly organized only in adulthood, physiological conditions can be evaluated and disorders excluded. In addition, performance tests are used to determine parameters for regulating training (e.g., heart rate, lactate thresholds). Nevertheless, despite apparent similar conditions and regular participation in training, performance developments differ between athletes. In the absence of performance or appearing complaints, diagnostic evaluations are initiated, not infrequently too late or with unremarkable results. During the season frequent medical follow-up examinations are not feasible. Therefore, simple diagnostic tools are necessary to characterize athletes’ status and well-being for ensuring an optimal performance development.

Based on these facts, a high performance capacity and an optimal health status are necessary requirements for a continuous progress and success. The aim is to determine valid predictors of performance development, which are easy to identify and apply in practice. High hemoglobin and vitamin D values are associated with an enhanced performance. Furthermore, hs-CRP may indicate an inflammatory process, and diminished performance and fatigue with concurrent unspecific flu-like symptoms are often combined with an Epstein Barr Virus (EBV) infection in competitive athletes (Gleeson et al., 2002; Balfour et al., 2015). Therefore, these potential predictors, in addition subjective health-related parameters, were recorded in a cohort of young competitive athletes with the purpose to determine whether these parameters can predict performance progress.

## Materials and Methods

### Participants

Individual performance developments of 146 young athletes (male [m]: *n* = 96 [65.8%], female [f]: *n* = 50 [34.2%]) of four different sports (cross-country skiing [CCS]: *n* = 35, cycling [CYC]: *n* = 48, soccer [SOC]: *n* = 45, swimming [SWI]: *n* = 18) were evaluated. With the exception of soccer, in each sport both genders were considered. Every exercise intervention period counted as one assessment, hence, the analyzes included possibly several examinations of one athlete. Data with missing values and/or inadequate details in recording were excluded. In total, 356 visits (= exercise intervention periods) were analyzed (mean age at visit 1 [V1]: 14.7 ± 1.7 years, gender distribution m/f: 57.6/42.4%, CCS: *n* = 77 [21.6%], CYC: *n* = 169 [47.5%], SOC: *n* = 81 [22.8%], SWI: *n* = 29 [8.1%]).

**FIGURE 1:**
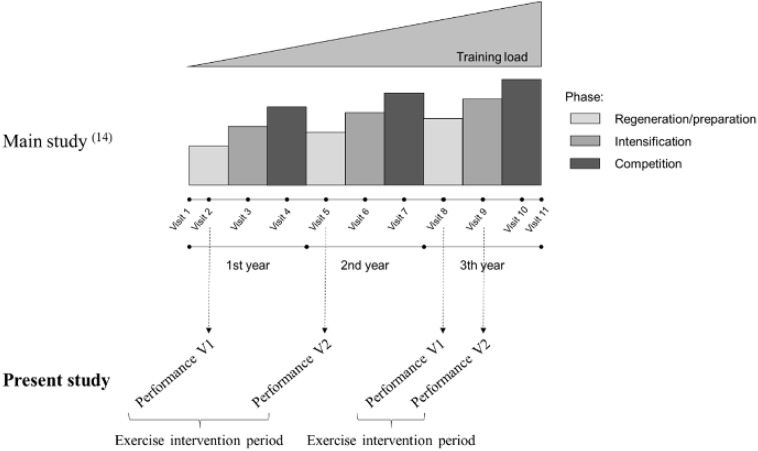
Study timeline (main and present study).

Athletes belonged to a controlled, prospective, longitudinal study, which was conducted between 2010 and 2014 (Blume et al., 2018). For individual characterization, participants (*n* = 274) were examined up to three times each year (during regeneration, training, and competitive season) regarding selected parameters to determine the effects of certain stress factors (e.g., training load), plus their dynamics, on chosen outcome measures (e.g., clinical, immunological, physiological, psychological, and performance end points). The main and present study timeline is shown in Figure 1.

### Sports

For the analyzes, sports were considered, which benefit from an improved endurance performance. In addition to classic endurance sports (cross-country skiing, cycling, swimming), soccer players were referred to the analyzes due to the knowledge that an aerobic endurance training improves individuals’ soccer performance (Helgerud et al., 2001). As a further study requirement, this sport is subject to a training macrocycle permitting the analyze of defined periods. Here, soccer players pass through weeks, which consist of specific endurance training. The focus of the study was the development of athletes’ endurance capacity. This has only a restricted value on sport-specific performance.

### Eligibility Criteria

Prior to commencement of the investigations, each athlete underwent a comprehensive clinical examination and were examined to assess inclusion (at V1 age ≤ 18 years, competing successfully at international or national level competitions for at least 2 years, belonging to training groups to ensure systematic training, future perspective of the athlete, written informed consent from parents and athletes) and exclusion criteria (e.g., chronic pathology and/or disability that affected their athlete’s career, long-lasting injury or illness at V1). For present analyzes, exclusion criteria have been extended: different performance diagnostic tests at V1 and V2 (e.g., due to illness/injury), not reaching the lactate thresholds (e.g., lack of motivation, achieving termination criteria such as ecg abnormalities), exceeding the examination periods. All subjects were fully informed about the rationale for the study and of all procedures to be undertaken. Before study participation, athletes and their parents signed a written informed consent form. The study was approved by the Medical Research Ethics Committee (TU Müenchen) and was in agreement with the principles outlined in the Declaration of Helsinki.

### Exercise Intervention Period

**FIGURE 2:**
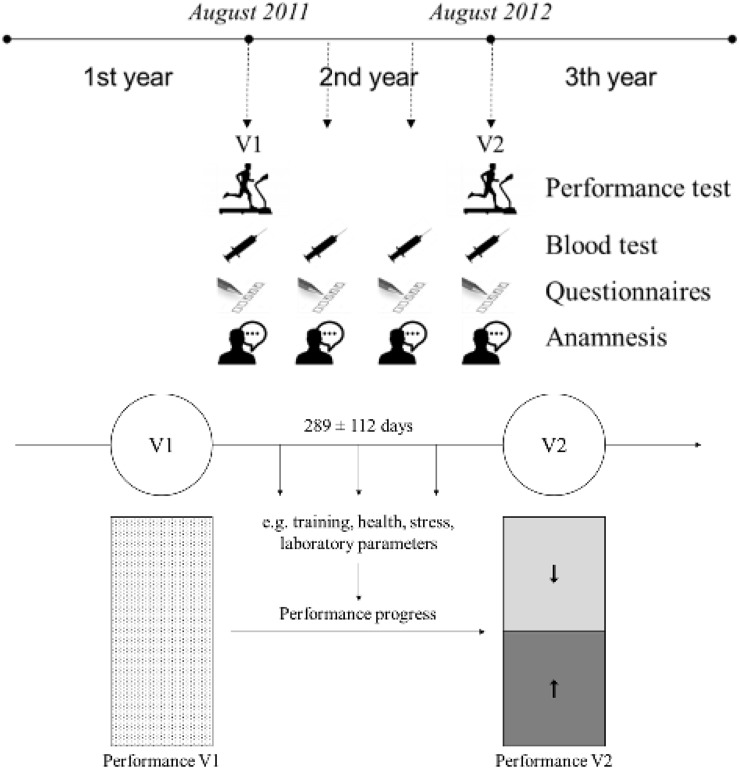
Study design: exercise intervention period. V1, Visit 1 (e.g., *SOC: August 2011*); V2, Visit 2 (e.g., *SOC: August 2012*).

Once a year each athlete underwent a comprehensive sports medical examination. Depending on the kind of sport, this investigation (V1) was scheduled at the beginning of the season (CCS: June, CYC: November, SOC: August, SWI: December), and was repeated, for monitoring, after one year (V2). Between V1 and V2, at defined examination times (interval: 4 months), selected parameters, regarding training, health, stress, performance, were collected (Figure 2). This observation period (V1-V2, long exercise intervention time) had the duration of 365 ± 22 days [d]. In addition, for assessing e.g., performance progress during the preparation (pre-season) period, certain sports (CCS, CYC) got more than one performance test per year (CCS: October, CYC: March). These short intervention periods (136 ± 40 d) accounted for 33.4% (*n* = 119) of the total analysis. In summary, athletes were prospectively followed for 289 ± 112 days.

### Determined Parameters

At V1, V2 and defined examination times between (Figure 2), selected parameters were collected. For this purpose, certain tools were used: anamnesis (medical history), questionnaires (training, health-related parameters, stress), blood draw, performance test. To evaluate the study end point (performance progress), individual performance parameters, in particular aerobic threshold, were measured, compared between V1 and V2, and the relative progress (Δ) calculated. To identify potential outcome-related predictors, selected parameters were analyzed: training hours (Th/w [h], Training hours per week), health sense (Hs [%]), stress level (Sl [%]), prevalences of recurrent infections (Ri [%]), feeling “unhealthy” (Fu [%]), fatigue (Fa [%]), and sleep disorders (Sd [%]), hemoglobin (Hb [g/dl]), high sensitivity C-reactive protein (hs-CRP [mg/dl]), vitamin D (VD [ng/ml], and Epstein-Barr-Virus serostatus (EBV [%]).

The used questionnaires were created for the study. Each athlete received a detailed verbally description.

#### Performance Progress (End Point)

Athletes performed before (V1) and after (V2) the exercise intervention period a standardized incremental test on a cycle ergometer (E) or a treadmill (T). Depending on gender and sport, initial load, incremental load, stage duration and incline differed. Exemplary, soccer players started the treadmill test (1% incline) with 6 kilometers per hours (km/h). Every three minutes, load was increased by 2 km/h until athletes’ exertion. Beside, swimmers performed the test on a cycle ergometer with an initial load of 50 Watt (W) and an incremental load of 30 W. Test protocols were chosen depending on sport-specific demands and were predetermined by national sports federations (SOC: T, 6 km/h [=initial load], 2 km/h [=incremental load], 3 min [=stage duration], 1% [=incline]; CCS male: T, 8 km/h, 1 km/h, 3 min, 5%; CCS female: T, 6 km/h, 1 km/h, 3 min, 5%; SWI: E, 50 W, 30 W, 3 min; CYC male: E, 80 W, 20 W, 3 min; CYC female: E, 60 W, 20 W, 3 min).

Participants were instructed to avoid intensive physical training 24 h prior the test. At defined times, before, during and after the test, selected parameters were assessed: heart rate, blood pressure, blood lactate (capillary blood samples from earlobe), and rating of perceived exertion (RPE). After analyzing the lactate concentrations, lactate thresholds were calculated. To determine the individual aerobic performance (P_aerob_), two fixed lactate thresholds were used (E: 3 mmol/l, T: 4 mmol/l), and declared as performance output, relative in watt per kilogram (E: W/kg), or rather, absolute in kilometers per hour (T: km/h) (Heck et al., 1985). Finally, individual thresholds between V1 and V2 were compared. The calculated differences were defined as performance progresses (Δ [%]) representing the study end point. For analyzes, performance development was categorized into two groups: impairment and improvement. An impairment corresponded with a difference (Δ) of 0% or lower (performance V2 = / < V1), an improvement of more than 0% (performance V2 > V1).

#### Potential Outcome-Related Parameters

##### Training hours

As part of every visit, individuals’ training hours per week (average number) were recorded using standardized questionnaires, training logs and interviews. In addition to current data, the average number of training hours per week (Th/w) of the last four ones were reported. For analyzes, the mean number of Th/w, evaluated between V1 and V2, was used. Furthermore, beside metric evaluation, training hours were categorized into three groups same size for each gender (tertiles [T]).

##### Health sense/stress level

**FIGURE 3:**
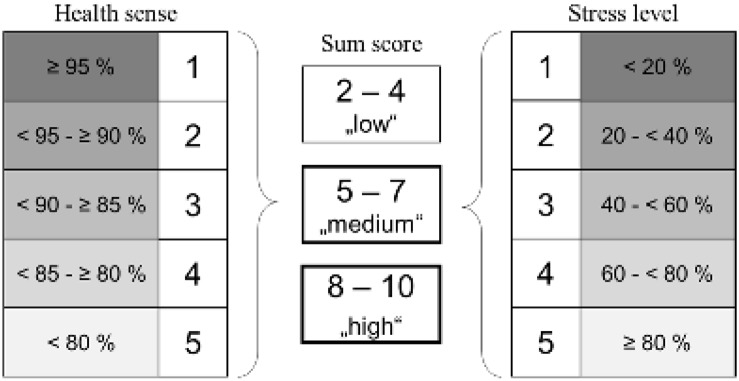
Categorizations of health sense (Hs) and stress level (Sl) to calculate sum score.

To evaluate subjective sensitivities, directly prior every visit, athletes were required to complete a questionnaire regarding, amongst others, subjective health sense (Hs) and stress level (Sl). The answers should reflect the condition of the last 4 weeks. Here, visual analog scales (VAS) were used, with a range between 0 and 100 percentages. High scores indicated an elevated stress level or rather an improved health sense (0%: “no stress”/”ill”, 100%: “highest stress level”/”healthy”). Athletes marked subjective feelings with a cross. For analyzes, mean values of health senses and stress levels, determining during V1 and V2, were applied. In addition, both parameters were categorized into five groups reflecting ordinal gradation (1: high health sense/low stress level, 5: low health sense/high stress level). Based on that, a sum score was calculated (Figure 3).

##### Other health-related parameters

Using a questionnaire, additional health-related parameters were requested: recurrent infections/susceptibility to infections, feeling “unhealthy”, fatigue, and sleep disorders. For that, athletes were asked if they felt more often sick (compared to the past/to others), felt unhealthy, suffered from fatigue (prolonged tiredness), and whether sleep disorders existed. The questions could be answered with “*yes*”, “*no*”, “*I don’t know*.” When responding with “*yes*” at least once between V1 and V2, athletes were categorized as clinically noticeable. Finally, based on this, prevalences were determined.

##### Laboratory parameters

Beside subjective information, biochemical data were determined at each examination. Thus, blood samples were taken standardized. All blood collections were obtained from the antecubital vein. Regarding performance, selected parameters were selected: hemoglobin concentration ([Hb], g/dl), high sensitivity C-reactive protein (hs-CRP, mg/dl), vitamin D (VD, ng/ml) and Epstein-Barr-Virus serostatus (EBV). All values were considered and mean values calculated. In addition, prevalence of EBV positive athletes was detected [detailed methods description in previous publication (Blume et al., 2018)].

Rationales of the selected biochemical parameters:

Hemoglobin: There exists a strong relationship between hemoglobin concentration ([Hb]), hemoglobin mass (Hb_mass_), maximal oxygen uptake (VO_2__max_) and, hence, endurance performance (Gore et al., 1997; Heinicke et al., 2001; Jacobs et al., 2011). So, studies demonstrated an increase of VO_2__max_ by 3.6 ml/min per 1 g hemoglobin (Prommer et al., 2018). Furthermore, low hemoglobin concentrations indicate anemia, which can negatively affect athletes’ performance (Schumacher et al., 2002; Novack et al., 2007). Because of the simple and low-cost assessment, [Hb] was used to examine its dependence on athletes’ performance. It was assumed that high [Hb] values correlate with a better outcome.
High-sensitivity C-reactive protein: CRP is a general marker for inflammation and infection, moreover, increased high-sensitivity CRP (hs-CRP) values reflect a low-grade systemic inflammation (Mazurek et al., 2011). Exercise-induced muscle damage and airway inflammation, which can negatively affect athletes’ training and performance, can induce an elevation of hs-CRP (Kasapis and Thompson, 2005; Ko et al., 2016). Therefore, it was assumed that high hs-CRP levels are associated with a poor performance development because of potentially underlying disorders.
Vitamin D: High vitamin D supplies are associated with an enhanced performance, furthermore, low values are discussed as a potentially limiting factor (Cannell et al., 2009; Dahlquist et al., 2015). Regarding this, vitamin D deficiency can negatively affect athletes’ health (Cannell et al., 2009; Ogan and Pritchett, 2013). Therefore, we examined the association between vitamin D values and athletes’ performance development.
Epstein Barr Virus: EBV is often associated with a diminished performance and fatigue in competitive athletes (Gleeson et al., 2002; Balfour et al., 2015). There has been an ongoing controversy, whether elite athletes suffer a higher rate of EBV infections, which can negatively affect athletes’ health and performance (Farrokhyar et al., 2015). It was assumed that athletes with a positive EBV serostatus showed lower performance developments.

### Statistical Analyzes

The data were compiled using Microsoft Excel^®^ and evaluated using the SPSS^®^ software package (version 25.0; SPSS Lead Technologies Inc., Chicago, IL, United States). Frequency distributions of all continuous variables were examined to detect outlying values, and the Kolmogorov-Smirnov test was used to check the normal distribution of variables. All results, assuming normal distribution, were presented as mean ± standard deviation (SD). Differences between groups were analyzed using an independent samples *t*-test. To determine the differences in the group analysis, ANOVA was used. The chi-square test was performed to verify possible differences between nominal scaled variables. Significance was accepted at the *p* < 0.05 level. Depending on the analysis, data were stratified by gender and sport, were presented by tertiles, and variables were categorized in ordinal gradation. All tables and graphics were generated with Microsoft Excel^®^ and SPSS^®^.

## Results

### Basic Characteristics

#### Participants

Individual performance developments of 146 young athletes (m: *n* = 96 [65.8%], f: *n* = 50 [34.2%]) of four different sports (SOC: *n* = 45, CYC: *n* = 48, SWI: *n* = 18, CCS: *n* = 35) were evaluated. In total, 356 exercise intervention periods were considered (Table 1). At V1 adolescent competitive athletes showed a mean age of 14.7 ± 1.7 years (m: 14.2 ± 1.7 years, f: 15.4 ± 1.4 years, *p* < 0.001), at V2 of 15.5 ± 1.8 years. Athletes were prospectively followed for 289 ± 112 days.

**TABLE 1:** Basic characteristics of the total athletes’ collective and dependent on sport and gender.

		***n* (%)**	**Age V1 [yrs]**	**Th/w [h]**	**Duration [d]**	**P_aerob_ V1**	**P_aerob_ V2**	**Δ [%]**
Total		356 (100)	14.7 ± 1.7	12.4 ± 4.6	289 ± 112			
	m	205 (57.6)	14.2 ± 1.7	11.2 ± 4.3	313 ± 98			
	f	151 (42.4)	15.4 ± 1.4	13.9 ± 4.6	256 ± 123			
	p^1^		<0.001^∗∗^	<0.001^∗∗^	<0.001^∗∗^			
SOC	m	81 (22.8)	13.8 ± 1.4	9.6 ± 2.8	362 ± 19	13.2 ± 1.4 km/h	14.1 ± 1.1 km/h	7.0 ± 10.0
CYC	m	71 (19.9)	13.6 ± 2.0	12.2 ± 3.8	245 ± 118	3.7 ± 0.5 W	3.9 ± 0.5 W	6.0 ± 11.4
	f	98 (27.5)	15.2 ± 1.4	14.2 ± 4.5	233 ± 123	3.1 ± 0.5 W	3.3 ± 0.5 W	5.6 ± 11.6
	p^1^		<0.001^∗∗^	=0.005^∗^	=0.529^ns^	<0.001^∗∗^	<0.001^∗∗^	=0.834^ns^
SWI	m	16 (4.5)	14.7 ± 1.6	14.2 ± 8.1	373 ± 24	2.8 ± 0.5 W	2.8 ± 0.6 W	1.1 ± 18.3
	f	13 (3.7)	15.1 ± 1.0	17.0 ± 4.5	377 ± 16	2.7 ± 0.5 W	2.7 ± 0.5 W	0.9 ± 8.1
	p^1^		= 0.443^ns^	=0.276^ns^	=0.652^ns^	=0.485^ns^	=0.565^ns^	=0.968^ns^
CCS	m	37 (10.4)	15.7 ± 1.2	10.6 ± 3.7	309 ± 99	12.5 ± 1.0 km/h	12.7 ± 1.0 km/h	2.2 ± 6.8
	f	40 (11.2)	16.1 ± 1.4	11.7 ± 3.7	273 ± 117	11.1 ± 0.6 km/h	11.2 ± 0.8 km/h	0.9 ± 5.8
	p^1^		=0.153^ns^	=0.295^ns^	=0.152^ns^	<0.001^∗∗^	<0.001^∗∗^	=0.388^ns^
p^2^			<0.001^∗∗^	<0.001^∗∗^	<0.001^∗∗^			=0.001^∗^

*V1, visit 1; V2, visit 2; SOC, soccer; CYC, cycling; SWI, swimming; CCS, cross-country skiing; m, male; f, female; yrs, years; Th/w, training hours per week; h, hours; d, days; P_*aerob*_, aerobic performance on lactate threshold of 3 mmol/l (E) or rather 4 mmol/l (T); Δ = delta P_*aerob*_ V1 vs. P_*aerob*_ V2; km/h, kilometers per hour; W, watt. Data are shown as mean ± SD and percentage values. *p*^1^, significance between genders; *p*^2^, significance between sports; ^∗∗^*p* < 0.001, ^∗^*p* < 0.01, ^#^*p* < 0.05, ^*ns*^*p* ≥ 0.05 (non-significant).*

#### Performance Development

In summary, athletes achieved a mean performance development of 4.7 ± 10.7%. Here, no gender differences were found (m: 5.3 ± 11.0%, f: 3.9 ± 10.3%, *p* = 0.238). Performance advances differed between the four sports (*p* = 0.001, Table 1). The highest relative performance progress reached soccer players (7.0 ± 10.0%), followed by male (6.0 ± 11.4%) and female cyclists (5.6 ± 11.6%). Table 1 represents all performance developments subdividing in dependence of sport and gender. 66.3% (*n* = 236) of all athletes showed an improvement of performance (Δ > 0%), 33.1% (*n* = 118) of at least 7.5%.

#### Potential Outcome-Related Predictors

Female athletes, compared to males, showed higher training loads per week (13.9 ± 4.6 h vs. 11.2 ± 4.5 h, *p* < 0.001), an impaired health sense (83.5 ± 11.1% vs. 87.5 ± 10.0%, *p* = 0.001), and elevated stress levels (50.1 ± 24.0% vs. 42.0 ± 23.1%, *p* = 0.003). During the intervention period, 15.0% complained recurrent infections, one in five athletes (20.7%) felt “unhealthy”, and 12.3% reported fatigue. Only 2.7% of all participants referred sleep disorders. Thereby, no gender differences were found. Male athletes possessed a mean hemoglobin value of 14.6 ± 0.9 g/dl, females of 13.5 ± 0.9 g/dl (*p* < 0.001). Almost two-thirds (64.0%) of all athletes was detected as EBV-positive, with a similar distribution between both genders (m: 65.9%, f: 61.6%, *p* = 0.407). Also, no gender differences in vitamin D levels (m: 33.2 ± 9.7 ng/ml, f: 37.0 ± 12.5 ng/ml, *p* = 0.168) and hs-CRP values (m: 0.096 ± 0.217 mg/dl, f: 0.077 ± 0.149 mg/dl, *p* = 0.452) were found. All potential outcome-related predictors dependent on sports are demonstrated in Table 2.

**TABLE 2:** Potential outcome-related predictors (training, subjective health-related, and biochemical parameters, stress) dependent on sport.

	**SOC**	**CYC**	**SWI**	**CCS**	***P***
**Training hours (Th/w)**	9.6 ± 2.8	13.3 ± 4.3	15.4 ± 6.8	11.1 ± 3.7	<0.001^∗∗^
**Stress level (%)**	42.0 ± 23.2	46.0 ± 24.8	49.1 ± 22.8	45.9 ± 22.3	=0.491^ns^
**Subjective health variables**					
Health sense (%)	89.3 ± 9.4	84.8 ± 11.2	86.0 ± 9.0	84.2 ± 10.7	=0.010^#^
Recurrent infections (%)	15.1	12.7	10.3	22.4	=0.263^ns^
Feeling “unhealthy” (%)	15.4	21.2	24.1	24.6	=0.545^ns^
Fatigue (%)	15.6	9.9	21.4	9.1	=0.241^ns^
Sleep disorders (%)	3.9	2.1	/	3.6	=0.668^ns^
**Biochemical markers**					
Hemoglobin (g/dl) m	14.3 ± 0.8	14.7 ± 1.0	14.6 ± 0.9	15.1 ± 0.7	<0.001^∗∗^
f	/	13.4 ± 0.7	13.3 ± 1.1	13.9 ± 1.0	=0.016^#^
hs-CRP (mg/dl)	0.094 ± 0.177	0.075 ± 0.140	0.065 ± 0.062	0.112 ± 0.292	=0.601^ns^
Vitamin D (ng/ml)	40.3 ± 8.9	31.4 ± 8.5	31.4 ± 16.5	38.0 ± 8.6	=0.015^#^
EBV-positive *n* (%)	71.6	46.7	75.9	89.6	<0.001^∗∗^

*SOC, soccer; CYC, cycling; SWI, swimming; CCS, cross-country skiing; m, male; f, female; Th/w, training hours per week. Data are shown as mean ±SD and percentage values. *p*, significance between sports; ^∗∗^*p* < 0.001, ^#^*p* < 0.05, ^*ns*^*p* ≥ 0.05 (non-significant).*

### Performance Improvement vs. Impairment

#### Training Hours, Sports

Athletes with a positive performance advance (Δ > 0%) trained similar loads weekly compared to unsuccessful participants during the intervention period (Δ > 0%: 12.1 ± 4.7 h, Δ ≤ 0%: 12.9 ± 4.5 h, *p* = 0.207, Table 3). Furthermore, soccer players offered the lowest number of training hours (9.6 ± 2.8 h, *p* < 0.001, Table 2), but showed the highest performance development (7.0 ± 10.0%, *p* = 0.001, Table 1). 76.5% of all soccer players demonstrated a positive performance advance (CYC: 68.6%, SWI: 55.5%, CCS: 54.5%, *p* = 0.014), 44.4% a development of minimum 7.5% (CYC: 40.8%, SWI: 31.0%, CCS: 14.3%, *p* = *0.001*). The parameter training hours showed sex-specific differences (Th: m: 11.2 ± 4.3 h, f: 13.9 ± 4.6 h, *p* < 0.001), therefore, this variable was categorized into three groups same size individually for each gender (m: Th1: ≤ 9 h, Th2: 9.1–12.7 h, Th3: ≥ 12.8 h; f: Th1: ≤ 12.5 h, Th2: 12.6–15.4 h, Th3: ≥ 15.5 h). Regarding this, performance development was unaffected by training load (m: Th1: 4.2 ± 9.7%, Th2: 5.8 ± 10.9%, Th3: 4.7 ± 12.3%, *p* = 0.748; f: Th1: 4.8 ± 10.6%, Th2: 6.2 ± 10.9%, Th3: 1.2 ± 10.4%, *p* = 0.092, Table 4).

**TABLE 3:** Potential outcome-related predictors (training, subjective health-related parameters, biochemical markers, stress) dependent on performance development.

**Aerobic performance**	**Δ ≤ 0% Impairment**	**Δ > 0% Improvement**	***p***
*n* (%)	120 (33.7)	236 (66.3)	
Training hours (Th/w)	12.9 ± 4.5	12.1 ± 4.7	=0.207^ns^
Health sense (%)	83.3 ± 10.7	87.2 ± 10.4	=0.001^∗^
Recurrent infections *n* (%)	20 (17.4)	29 (13.7)	=0.379^ns^
Feeling “unhealthy” *n* (%)	33 (30.3)	33 (15.7)	=0.002^∗^
Fatigue *n* (%)	18 (17.0)	19 (9.7)	=0.068^ns^
Sleep disorders *n* (%)	2 (1.9)	6 (3.1)	=0.535^ns^
Stress level (%)	50.8 ± 22.4	42.3 ± 24.0	=0.002^∗^
Hemoglobin (g/dl) m	14.6 ± 0.8	14.6 ± 1.0	=0.926^ns^
f	13.5 ± 0.9	13.6 ± 0.9	=0.578^ns^
hs-CRP (mg/dl)	0.124 ± 0.284	0.072 ± 0.132	=0.043^#^
Vitamin D (ng/ml)	33.9 ± 12.0	34.6 ± 9.9	=0.787^ns^
EBV-positive *n* (%)	76 (63.3)	152 (64.4)	=0.842^ns^

*Collective: total cohort. Δ = delta P_*aerob*_ V1 vs. P_*aerob*_ V2; Th/w, training hours per week. Data are shown as mean ± SD and percentage values. ^∗∗^*p* < 0.001, ^∗^*p* < 0.01, ^#^*p* < 0.05, ^*ns*^*p* ≥ 0.05 (non-significant).*

**TABLE 4:** Performance development dependent on potential outcome-related predictors (stress, health sense, training hours, subjective health-related parameters, biochemical markers).

Health sense (%)	≥ 95	≥90 to <95	≥ 85 to <90	≥80 to <85	<80	
Performance Δ (%)	7.6 ± 10.3	4.8 ± 12.7	5.5 ± 10.2	2.9 ± 8.8	2.4 ± 11.2	=0.040^#^

Stress level (%)	< 20	20 to <40	40 to <60	60 to <80	≥80	
Performance Δ (%)	7.1 ± 9.4	7.0 ± 11.0	3.3 ± 8.8	3.7 ± 11.5	1.8 ± 14.8	=0.045^#^

Hemoglobin (g/dl)	m: ≥15.0		m: 14.3 to 14.9		m: ≤14.2	
	f: ≥13.9		f: 13.3 to 13.8		f: ≤13.2	
Performance Δ (%)	5.1 ± 10.8		4.9 ± 12.8		4.2 ± 8.6	=0.772^ns^

hs-CRP (mg/dl)	≤0.026		0.027 to 0.058		≥0.059	
Performance Δ (%)	6.1 ± 8.8		5.3 ± 10.0		4.5 ± 11.8	=0.588^ns^

Vitamin D (ng/ml)	> 20				≤20	
Performance Δ (%)	4.9 ± 11.6				5.4 ± 23.7	=0.578^ns^

EBV serostatus	Negative				Positive	
Performance Δ (%)	5.4 ± 12.1				4.3 ± 9.8	=0.374^ns^

Training hours (Th/w)	m: ≤9.0		m: 9.1 to 12.7		m: ≥12.8	
	f: ≤12.5		f: 12.6 to 15.4		f: ≥15.5	
Performance Δ (%)	m: 4.2 ± 9.7		m: 5.8 ± 10.9		m: 4.7 ± 12.3	=0.748^ns^
	f: 4.8 ± 10.6		f: 6.2 ± 10.9		f: 1.2 ± 10.4	=0.092^ns^

*m, male; f, female; Th/w, training hours per week; Δ = delta P_*aerob*_ V1 vs. P_*aerob*_ V2. Data are shown as mean ± SD and percentage values. ^#^*p* < 0.05, ^*ns*^*p* ≥ 0.05 (non-significant).*

#### Subjective Health-Related Variables

**FIGURE 4:**
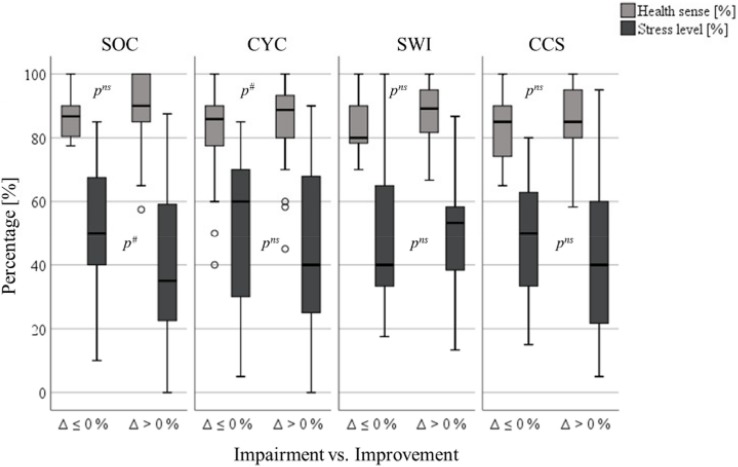
Sport-specific health senses and stress levels dependent on performance development. Δ = delta P_aerob_ V1 vs. P_aerob_ V2; SOC, soccer; CYC, cycling; SWI, swimming; CCS, cross-country skiing. ^#^*p* < 0.05, ^ns^*p* ≥ 0.05 (non-significant).

**FIGURE 5:**
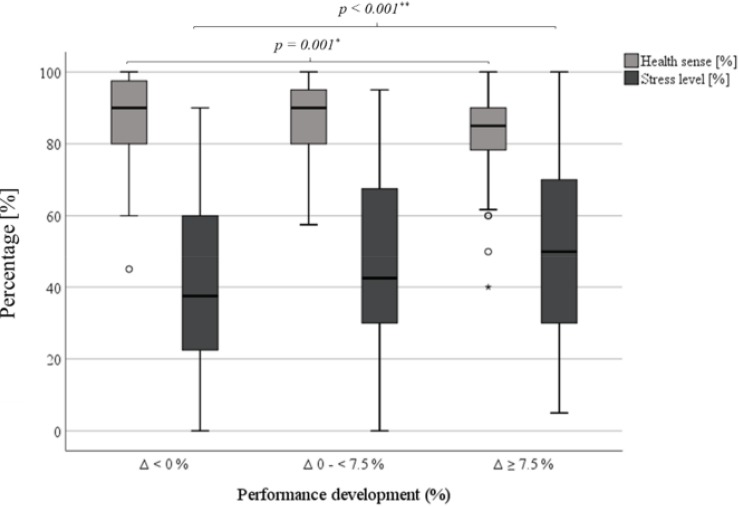
Health senses and stress levels dependent on performance development (total cohort). Δ = delta P_aerob_ V1 vs. P_aerob_ V2. ^∗∗^*p* < 0.001, ^∗^*p* < 0.01.

Athletes with a positive performance development reported higher health sense levels during the exercise intervention period compared to the group with an impairment (Δ > 0%: 87.2 ± 10.4%, Δ ≤ 0%: 83.3 ± 10.7%, *p* = 0.001, Table 3). This result was confirmed for cyclists (Δ > 0%: 86.1 ± 10.3%, Δ ≤ 0%: 82.1 ± 12.5%, *p* = 0.040, Figure 4). Similar tendencies were evident in the other groups, but without significance (SOC: *p* = 0.185, SWI: *p* = 0.247, CCS: *p* = 0.321, Figure 4). Health sense differences were detectable in both genders, particularly significant among female athletes (m: Δ > 0%: 88.4 ± 10.4%, Δ ≤ 0%: 85.4 ± 8.8%, *p* = 0.056; f: Δ > 0%: 85.3 ± 10.1%, Δ ≤ 0%: 80.7 ± 12.2%, *p* = 0.023). Furthermore, athletes with an improvement of at least 7.5% offered the highest subjective health senses (Δ ≥ 7.5%: 88.0 ± 10.5%, Δ 0 to <7.5%: 86.2 ± 10.2%, Δ < 0%: 83.3 ± 10.7%, *p* = 0.004, Figure 5). Notably, also significant differences among male athletes were demonstrated (Δ ≥ 7.5%: 89.8 ± 10.1%, Δ 0 to < 7.5%: 86.8 ± 10.5%, Δ < 0%: 85.3 ± 8.8%, *p* = 0.034). For further analyzes, health sense levels were divided into five groups. Here, performance developments increased with elevating health sense categorizes (*p* = 0.040, Table 4).

**FIGURE 6:**
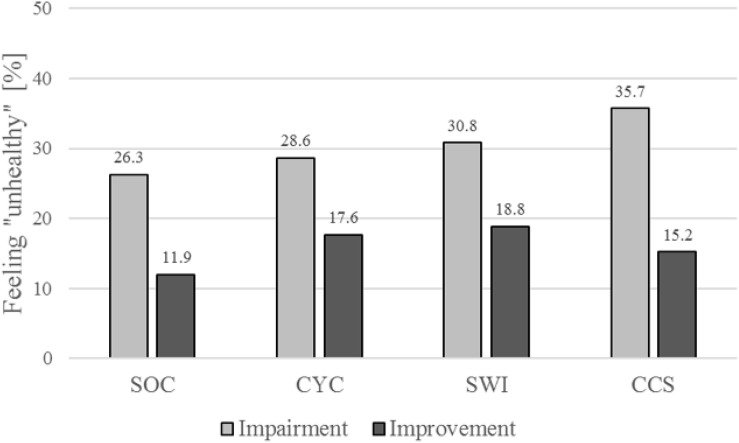
The subjective health-related parameter feeling “unhealthy” dependent on performance development and sport. SOC, soccer; CYC, cycling; SWI, swimming; CCS, cross-country skiing.

Compared to the group with an improved performance development, almost twice as many athletes reported a “unhealthy” feeling (Δ > 0%: 15.7%, Δ ≤ 0%: 30.3%, *p* = 0.002, Table 3, Figure 6). This difference was replicable among female athletes (Δ > 0%: 12.5%, Δ ≤ 0%: 35.3%, *p* = 0.002), in the male collective without significance (Δ > 0%: 17.7%, Δ ≤ 0%: 25.9%, *p* = 0.198).

Considering the prevalences of recurrent infections and fatigue, there were noticeably differences between the performance progress groups. So, athletes with an improvement reported fewer recurrent infections (13.7% vs. 17.4%) and less fatigue (9.7% vs. 17.0%), but without significance (Ri: *p* = 0.379, Fa: *p* = 0.068, Table 3). However, in the group of cyclist a significant difference of reported recurrent infections was found (Δ > 0%: 8.4%, Δ ≤ 0%: 22.0%, *p* = 0.017). In contrast, there was no relationship between the outcome performance advance and the parameter sleep evident (Table 3).

#### Stress Level

**FIGURE 7:**
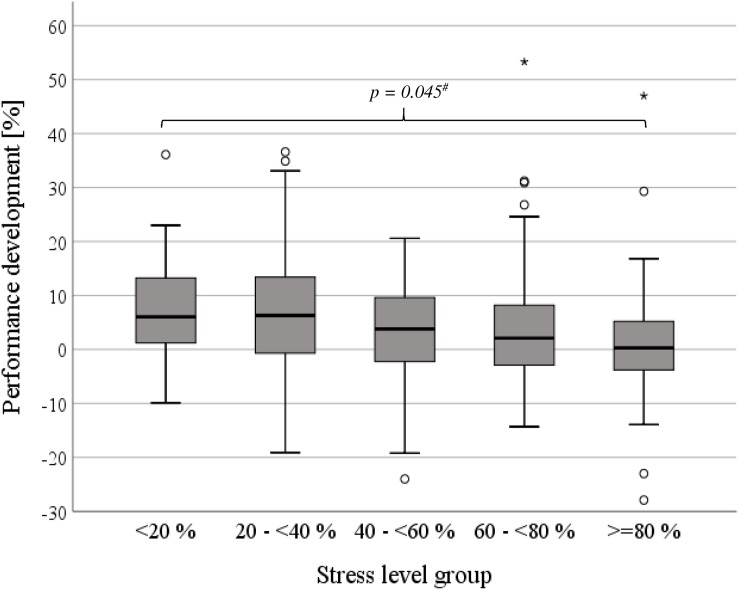
Performance development dependent on stress level group. ^#^*p* < 0.05.

Athletes with an impaired performance development felt more stressed during the exercise intervention period compared to them with a positive advance (Δ > 0%: 42.3 ± 24.0%, Δ ≤ 0%: 50.8 ± 22.4%, *p* = 0.002, Table 3). This result was confirmed for soccer players (Δ > 0%: 39.0 ± 23.0%, Δ ≤ 0%: 51.1 ± 22.1%, *p* = *0.048*, Figure 4). Similar tendencies were evident in the other groups, but without significance (CYC: *p* = 0.063, SWI: *p* = 0.956, CCS: *p* = 0.119, Figure 4). Furthermore, with increase in performance, a less extent of stress levels was observed (Δ ≥ 7.5%: 40.0 ± 23.1%, Δ 0 to <7.5%: 45.1 ± 24.5%, Δ < 0%: 51.0 ± 22.6%, *p* = 0.002, Figure 5). After stress level categorization into five groups, an increasing performance development with declined stress levels were observed (*p* = 0.045, Table 4, Figure 7). Here, an improved performance development prevalence (Δ > 0%) was found comparing the highest and lowest stress level category with each other (*p* = 0.014, Figure 7).

#### Biochemical Markers

There were no differences of hemoglobin values between athletes with performance improvements and impairments (m: Δ > 0%: 14.6 ± 1.0 g/dl, Δ ≤ 0%: 14.6 ± 0.8 g/dl, *p* = 0.926; f: Δ > 0%: 13.6 ± 0.9 g/dl, Δ ≤ 0%: 13.5 ± 0.9 g/dl, *p* = 0.578, Table 3), in all sports (SOC: *p* = 0.086; CYC: m: *p* = 0.060, f: *p* = 0.363; SWI: m: *p* = 0.600, f: *p* = 0.933; CCS: m: *p* = 0.211, f: *p* = 0.571). Furthermore, in both genders, also a high performance progress was not associated with higher hemoglobin values (m: Δ ≥ 7.5%: 14.6 ± 0.9 g/dl, Δ 0 to < 7.5%: 14.7 ± 1.0 g/dl, Δ < 0%: 14.6 ± 0.8 g/dl, *p* = 0.910; f: Δ ≥ 7.5%: 13.5 ± 0.9 g/dl, Δ 0 to < 7.5%: 13.7 ± 0.9 g/dl, Δ < 0%: 13.5 ± 0.9 g/dl, *p* = 0.827). For further analyzes, hemoglobin values were divided into three groups (ordinal gradation). Here, performance advances were similar between the groups (*p* = 0.772, Table 4).

Also, vitamin D values (Δ > 0%: 34.6 ± 9.9 ng/ml, Δ ≤ 0%: 33.9 ± 12.0 ng/ml, *p* = 0.787, Table 3) and EBV-serostatus (Δ > 0%: 64.4%, Δ ≤ 0%: 63.3%, *p* = 0.842, Table 3) showed no noticeably differences between the performance development groups in the total collective. As an exception, cross-country skiers with a performance improvement offered higher vitamin D values (Δ > 0%: 46.1 ± 3.5 ng/ml, Δ ≤ 0%: 33.2 ± 6.7 ng/ml, *p* = 0.022). This result was not found in the other groups (SOC: *p* = 0.510, CYC: *p* = 0.445, SWI: *p* = 0.910). Vitamin D values below 20 ng/ml are defined as deficiency. Based on this cut-off value, 8.2% of all athletes offered too low levels. However, this group showed no impaired performance progress (VD ≤ 20 ng/ml: 5.4 ± 23.7%, VD > 20 ng/ml: 4.9 ± 11.6%, *p* = 0.578, Table 4). EBV-positive athletes improved their performance by 4.3 ± 9.8% (m: 4.9 ± 10.4%, f: 3.6 ± 8.9%), seronegative youths by 5.4 ± 12.1% (m: 6.1 ± 12.0%, f: 4.5 ± 12.3%), in any case without significance (total: *p* = 0.374, m: *p* = 0.426, f: *p* = 0.608, Table 4).

In athletes with an improvement of performance lower hs-CRP values were measured (Δ > 0%: 0.072 ± 0.132 mg/dl, Δ ≤ 0%: 0.124 ± 0.284 mg/dl, *p* = 0.043, Table 3). This result was found in the group of soccer players (Δ > 0%: 0.064 ± 0.097 mg/dl, Δ ≤ 0%: 0.204 ± 0.314 mg/dl, *p* = 0.003), but not in the other sports (CYC: *p* = 0.488, SWI: *p* = 0.261, CCS: *p* = 0.328). However, there was no dependence regarding the extent of the positive performance progress (Δ ≥ 7.5%: 0.082 ± 0.164 mg/dl, Δ 0 to < 7.5%: 0.061 ± 0.085 mg/dl, Δ < 0%: 0.125 ± 0.286 mg/dl, *p* = 0.090). For further analyzes, the values of hs-CRP, in ascending order, were categorized into three groups. There were no effects on performance progress, neither after comparison of all three groups (hs-CRP1: 6.1 ± 8.8%, hs-CRP2: 5.3 ± 10.0%, hs-CRP3: 4.5 ± 11.8%, *p* = 0.588, Table 4), nor between group hs-CRP1 and hs-CRP3 (*p* = 0.310).

### Interaction Between Training Hours, Health Senses, Stress Levels, Other Health-Related Parameters and Biochemical Markers

Decreased subjective health senses were associated with elevated stress levels (Hs < 80%: 54.4 ± 19.9%, Hs ≥ 95%: 33.4 ± 25.2%, *p* < 0.001). Also, an impaired health sense, in particular values below 80%, was combined with higher prevalences of recurrent infections (Hs < 80%: 36%, Hs ≥ 95%: 2.7%, *p* < 0.001), fatigue (Hs < 80%: 28.8%, Hs ≥ 95%: 2.8%, *p* < 0.001), and the feeling “unhealthy” (Hs < 80%: 61.9%, Hs ≥ 95%: 0%, *p* < 0.001). In contrast, there were no significant differences of hs-CRP values (Hs < 80%: 0.143 ± 0.343 mg/dl, Hs ≥ 95%: 0.087 ± 0.185 mg/dl, *p* = 0.168) and vitamin D levels (Hs < 80%: 31.9 ± 12.4 ng/ml, Hs ≥ 95%: 31.4 ± 9.2 ng/ml, *p* = 0.067) between the groups. Beside health senses, stress levels showed no associations to the other health-related parameters (Ri: *p* = 0.295; Fu: *p* = *0.139*; Fa: *p* = 0.145). However, just stress levels of less than 20% were linked with moderate prevalences (Ri: Sl < 20%: 9.3%, Sl ≥ 80%: 19.2%; Fu: Sl < 20%: 9.3%, Sl ≥ 80%: 22.2%; Fa: Sl < 20%: 7.7%, Sl ≥ 80%: 11.1%). The parameter training hours showed sex-specific differences (Th: m: 11.2 ± 4.3 h, f: 13.9 ± 4.6 h, *p* < 0.001), therefore, this parameter was categorized into three groups same size individually for each gender (m: Th1: ≤ 9 h, Th2: 9.1–12.7 h, Th3: ≥ 12.8 h; f: Th1: ≤ 12.5 h, Th2: 12.6 – 15.4 h, Th3: ≥ 15.5 h). Regarding these classifications, neither stress levels (m: Tl1: 39.8 ± 24.9%, Tl2: 47.7 ± 22.0%, Tl3: 42.2 ± 19.6%, *p* = 0.187; f: Tl1: 49.0 ± 20.9%, Tl2: 50.9 ± 24.6%, Tl3: 52.0 ± 26.6%, *p* = 0.850) nor health senses (m: Tl1: 88.5 ± 9.9%, Tl2: 84.7 ± 10.2%, Tl3: 88.4 ± 8.6%, *p* = 0.074; f: Tl1: 82.8 ± 9.3%, Tl2: 86.3 ± 11.1%, Tl3: 83.6 ± 10.1%, *p* = 0.308) were dependent on extent of training load. Also, there were no prevalence differences of recurrent infections (m: Th1: 11.3%, Th2: 18.4%, Th3: 7.3%, *p* = 0.216; f: Th1: 11.4%, Th2: 18.9%, Th3: 29.5%, *p* = 0.101), feeling “unhealthy” (m: Th1: 20.7%, Th2: 27.7%, Th3: 18.2%, *p* = 0.495; f: Th1: 17.1%, Th2: 22.9%, Th3: 23.3%, *p* = 0.746), and fatigue (m: Th1: 14.5%, Th2: 8.9%, Th3: 18.4%, *p* = 0.416; f: Th1: 8.1%, Th2: 17.6%, Th3: 11.6%, *p* = 0.467).

### Sum Score Health Sense/Stress Level

**FIGURE 8:**
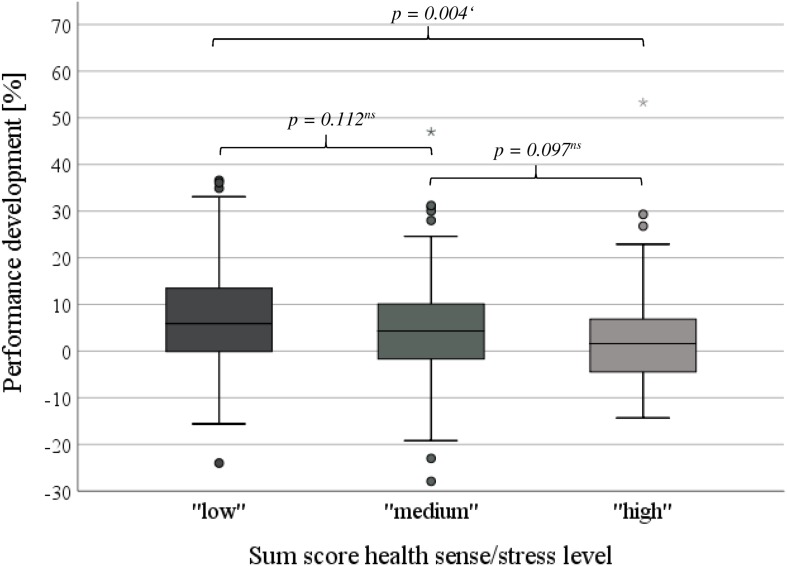
Performance development dependent on sum of health sense and stress level group (“low” [sum 2–4] vs. “medium” [sum 5–7] vs. “high” [sum 8–10] risk). ^∗∗^*p* < 0.001, ^∗^*p* < 0.01, ^#^*p* < 0.05, ^ns^*p* ≥ 0.05 (non-significant).

Because of their significant impact on performance development and their interaction each other, a sum score of health sense and stress level was formed. This score was based on the group numbers (1–5) with a range between 2 and 10 (Figure 3). Considering the sum score values, three groups were categorized: “low” (sum 2–4, high health sense/low stress level), “medium” (sum 5–7), and “high” risk (sum 8–10, low health sense/high stress level). The “low” risk group possessed a mean health sense of 95.1 ± 4.5% and a mean stress level of 22.4 ± 14.0%, the “high” risk group of 74.3 ± 7.3%, respectively 66.7 ± 12.3% (Table 5). In summary, 44.5% of all athletes demonstrated scores between 5 and 7 (“medium” risk), almost one quarter (23.7%) belonged to the “high” risk group. The distribution pattern differed significantly between male and female athletes (m/f: “low” 39.4/20.9%, “medium” 39.9/51.2%, “high” 20.7/27.9%, *p* = 0.002). There were no differences in training hours (“low” 12.2 ± 5.0 h, “medium” 12.8 ± 4.3 h, “high” 12.0 ± 4.7 h, *p* = 0.839, Table 5) between the three groups. In contrast, significant distinctions of the other health-related parameters were found (Table 5). Comparing the “high” with the “low” risk group, the prevalence of recurrent infections was fivefold (“low” 5.2%, “high” 26.0%, *p* < 0.001), furthermore, nearly tenfold of feeling “unhealthy” (“low” 5.0%, “high” 48.0%, *p* < 0.001) and twice as much of fatigue (“low” 2.2%, “high” 4.2%, *p* < 0.001). For all applicable, an ordinal increase in prevalence was evident (Table 5). Finally, performance development diminished in dependence of group affiliation (“low” 6.9 ± 10.9%, “medium” 4.7 ± 10.8%, “high” 2.1 ± 10.9%, *p* = 0.015, Figure 8).

**TABLE 5:** Potential outcome-related predictors (training, stress, subjective health-related parameters) and performance development dependent on sum of health sense and stress level group (sum 2–10).

**Health sense + Stress level (2–10)**	**2, 3, 4 “low”**	**5, 6, 7 “medium”**	**8, 9, 10 “high”**	***p*^a^**	***p*^b^**
%	31.9	44.5	23.7		
Training load (Th/w)	12.2 ± 5.0	12.8 ± 4.3	12.0 ± 4.7	=0.433^ns^	=0.858^ns^
Health sense (%)	95.1 ± 4.5	85.5 ± 8.8	74.3 ± 7.3	<0.001^∗∗^	<0.001^∗∗^
Stress level (%)	22.4 ± 14.0	50.1 ± 20.1	66.7 ± 12.3	<0.001^∗∗^	<0.001^∗∗^
Recurrent infections (%)	5.2	16.8	26.0	=0.001^∗^	<0.001^∗∗^
Feeling “unhealthy” (%)	5.0	17.0	48.0	<0.001^∗∗^	<0.001^∗∗^
Fatigue (%)	2.2	13.1	23.6	<0.001^∗∗^	<0.001^∗∗^
Sleep disorders (%)	2.2	2.2	4.2	=0.651^ns^	=0.459^ns^
Performance development (%)	6.9 ± 10.9	4.7 ± 10.8	2.1 ± 10.9	=0.015^#^	=0.004^∗^

*Th/w, training hours per week. Data are shown as mean ± SD and percentage values. ^*a*^significance between all three groups, ^*b*^significance between group “low” (sum 2–4) and “high” (sum 8–10) risk. ^∗∗^*p* < 0.001, ^∗^*p* < 0.01, ^#^*p* < 0.05, ^*ns*^*p* ≥ 0.05 (non-significant).*

## Discussion

Systematic training is an indispensable requirement for the development of a successful performance. However, similar training modalities, such as achieved training hours, lead to individual responses, and finally, decide on athletes’ success or failure. To foresee performance development, potential predictors should be known. For that, the evaluation of subjective parameters, in addition to known performance-related variables, such as biochemical markers, was focus of the analysis.

To increase individual performance over time, sufficient training loads are necessary. Competitive sport is associated with frequent, prolonged and intensive training sessions, even at a young age. 13-year-old or younger athletes in several sports train 15–20 h per week (Armstrong and McManus, 2011). Due to the earlier professionalization (e.g., *Youth Olympic Games*, *European Youth Olympics Festival*), the danger of a premature training cumulation is obvious (Myer et al., 2015). These physical strains can be linked with an increased risk for illness and injury (Hastmann-Walsh and Caine, 2015). Consistently, physical stress activates the immune system more or less, resulting in weakness or stabilization. However, the clinical relevance of such immunological changes, triggered by exercise, remains controversial, because no direct associations to increased infection rates could be clearly demonstrated (Fricker et al., 2005; Cox et al., 2008). Furthermore, it is unclear, from which load (e.g., duration, intensity, frequency) the immune system is negatively influenced, from when clinical complaints occur, and therefore, the performance development will be diminished (Konig et al., 2000). The average training load of the analyzed athletes was nearly 12 h per week, with a maximum of 30 h. The results presented an unaffected performance development by number of training hours. Here, the impairment and improvement groups showed similar amounts of mean training hours weekly. In addition, soccer players, who offered the lowest training hours, demonstrated the highest performance advances. Nevertheless, besides the quantitative evaluation of training loads, quality (e.g., intensity, type of training) should be considered as well. Too low individual intensities induce missing impulses, and thus, avoid required adaptations. In contrast, excessive intensities can negatively affect recovery and health causing frequent interruptions and lack or stagnation of performance. Therefore, training loads should be individually determined and adjusted regularly. In addition, the initial performance status should be taken into account, because performance and relative development show no linear relationship to each other. So, weaker athletes demonstrate possible greater performance improvements in less time. In summary, for further analyzes the inclusion of training quality, the evaluation of basic values (requirement of standardized performance diagnostic tests) and the assessment of detailed objective training logs (e.g., by digital data transfer) is recommended. The results are emphasized by a lack of dependence of neither subjective stress levels, health senses, nor of the occurrences of clinical complaints on extent of training load. In adolescence, overall strains of 60 h per week, including schooldays, may be present. Hence, perceived increased stress levels can have a negative impact on health (Main et al., 2010; Kellmann et al., 2018). The detected missing relation between training load and stress confirm previous published data (Blume et al., 2018). Here, athletes demonstrated similar stress levels despite higher training loads compared to a control group. This illustrates the individual and multi-factorial etiology of stress, the discrepancy among existing and perceiving stress, respectively, the individuals’ handling with stress factors. In future analyzes, possible further triggering factors regarding stress (e.g., psychological parameters) should be identified and analyzed. Despite this, results can be based on known positive effects of exercise on stress sense (e.g., vagotonic increase, structured everyday life, social environment, recognition, mental stability) and on the young age of the collective (e.g., less pressure, “playful” component) (Blume et al., 2018). These assumptions require the examination of adult elite athletes using a similar study design. In summary, the parameter training hours possessed no negative influence regarding athletes’ performance and health.

The aim of an exercise intervention, respectively of a pre-season period, is to improve individual performance. In particular, in endurance athletes, performance is directly associated with a high maximum oxygen consumption (VO_2__max_) (Bassett and Howley, 2000; Mairbäurl, 2013). Beside oxygen utilization, one key factor of a high VO_2__max_ is an enhanced oxygen transport capacity accomplished by hemoglobin (Wagner, 1996). Previous studies proved the predictive value of a high hemoglobin mass in endurance sports regarding performance (Steiner et al., 2019; Zelenkova et al., 2019). In contrast, most athletes demonstrate normal hemoglobin concentrations because of plasma volume changes, so called hemodilution (Schumacher et al., 2002). Therefore, an evident relationship between concentration values and competitive success is missing (Kuipers et al., 2007). Nevertheless, in clinical practice, an assessment of the hematological profile, notably of hemoglobin concentrations, is used to estimate and predict athletes’ performance, and in particular to detect disorders such as anemia. The results showed gender-specific differences of hemoglobin concentrations, but at no time an association with the endpoint performance development. Here, athletes with significant performance improvements (≥7.5%) offered similar values compared to the others, moreover, the lowest concentrations had none negative impact on progress. Furthermore, athletes with diminished hemoglobin concentrations (<12 g/dl, prevalence 2.2%) demonstrated no enlarged performance loss. Thus, present results are consistent with previous studies (Kuipers et al., 2007). Regular monitoring of hematological parameters is still recommended, not for the prediction of performance, but to detect abnormalities such as anemia. At once, further parameters should be considered, especially the evaluation of iron metabolism (e.g., ferritin), because a non-anemic iron depletion may impair performance (DellaValle and Haas, 2011).

In the last decade, vitamin D has been given special attention. Low values are discussed as a potentially performance limiting factor, in contrast, high vitamin D supplies are associated with an enhanced performance (Cannell et al., 2009; Dahlquist et al., 2015). Furthermore, a vitamin D deficiency can negatively affect athletes’ health (Cannell et al., 2009; Ogan and Pritchett, 2013). The importance regarding athletes performance remains uncertain, because placebo-controlled studies showed no improved physical performance of athletes with raised vitamin D serum concentrations (Dubnov-Raz et al., 2015; Fairbairn et al., 2018). Confirming this, the present analysis demonstrated no consistent relationship between vitamin D values and performance progress. Only in the group of cross-country skiers, an elevated vitamin D value was associated with an improved performance development. Beside this, none impaired outcome of vitamin D deficient athletes were found (Dubnov-Raz et al., 2015; Fairbairn et al., 2018). Compared to the literature, the prevalence of deficiency was low (Farrokhyar et al., 2015). Finally, prospective, controlled studies are required to evaluate long-term effects of a chronic vitamin D deficiency in young age.

In contrast to hemoglobin and vitamin D, Epstein Barr Virus (EBV) is often associated with a diminished performance and fatigue in competitive athletes (Gleeson et al., 2002; Balfour et al., 2015). There has been an ongoing controversy, whether elite athletes suffer a higher rate of EBV infections (Farrokhyar et al., 2015). Furthermore, previous studies interpreted slightly elevated EBV-specific IgG titers over the competition season as a reaction to increased EBV activity, possibly inducing an increased susceptibility to infections and an impaired performance (Pottgiesser et al., 2006; Farrokhyar et al., 2015). The results showed no dependence of performance progress on EBV serostatus. Moreover, EBV positive athletes demonstrated similar performance developments compared to seronegative participants. This is consistent with our previous publication, in which young elite athletes presented no different EBV-specific serological parameters compared to controls (Blume et al., 2018). Also, no direct relationships between training loads, clinical complaints, and EBV-specific immune responses (e.g., extent of IgG titers) were found (Blume et al., 2018).

Elite athletes are exposed to high strains, not only physically. The known stress factors include e.g., psychological pressure (e.g., annual selection, performing in competition), unstable financial support, training and competition environment factors (e.g., weather, inadequate training facilities), travel, nutrition, mismatch between internal and external expectations, protracted injuries, and repeated illnesses (Sabato et al., 2016). In young age, also puberty, a changed physical development, school stress, surrounding conflicts (with e.g., parents, friends, coach, and teachers) and an upcoming prioritization (less leisure time) should not be underestimated (Armstrong and Mc Manus, 2011). In addition, the trend of recent years shows an increased duration, intensity, and difficulty of training, a high-frequency participation in sports events, and an earlier specialization and professionalization (Armstrong and Mc Manus, 2011). All these potential risk factors can negatively affect the risk of illness and injury (Armstrong and Mc Manus, 2011; Sabato et al., 2016). While exercise has various positive effects on athletes health and well-being (cardiovascular fitness, muscular strength, bone health, weight control, self-confidence stabilization, reduced morbidity) (Wartburton and Bredin, 2016, 2017), prolonged and intensive training, in competitive sports unavoidable, can diminish the immune competence with following higher rates of infections. Furthermore, recurrent infections can cause frequent interruptions, lack or stagnation of performance, up to retirement from competitive sport (Maffulli et al., 2010). However, the phenotypes of athletes differ significantly among each other: only a small percentage of junior athletes achieve senior level, participate internationally, or even win medals at Olympics. Others are permanent sick or injured, moreover, despite apparently similar conditions, are not able to access their real performance in competition. In this regard, recent studies have shown fewer episodes of infections in successful top-ranking athletes, potentially based on selection mechanisms of “talented” ones (Hellard et al., 2015; Schwellnus et al., 2016). Unfortunately, a comprehensive definition of “talented” athletes is lacking, therefore, an identification in young age represents still a huge challenge (Johnston et al., 2018). The analyzes showed a significant relationship between health sense, stress level and performance development. Here, poor subjective health senses or elevated stress levels, and moreover their combination, result to an impaired performance progress comparing to athletes without reported disturbances. Regardless of hemoglobin values, subjective stress levels were a key factor modifying performance. Training loads possessed none influence on subjective stress levels, therefore, in case of increased stress levels, an exact diagnostics of potential provoking risk factors should be occurred. Subsequently, trigger factors can be minimized by specific interventions.

Competitive athletes, in particular cyclists and cross-country skiers, requires superior physiological skills, such as a trained aerobic and anaerobic performance, and accordingly a high maximum oxygen uptake (Bell et al., 2017). For the analyzes, aerobic performance capacity (relative performance at 3 mmol/l, or rather 4-mmol/l lactate) as outcome was chosen. The decision for this parameter was based on its standardized determination in contrast to competition results, although a high aerobic performance capacity does not reflect individual overall performance and sport-specific skills. However, also an aerobic endurance training improves soccer performance (Helgerud et al., 2001). Furthermore, an enhanced endurance performance reduces athletes’ regeneration time resulting in training advantages (Kellmann et al., 2018). It still remains to be considered, that missing performance developments are based on differently selected training priorities. Nevertheless, the analyzed exercise intervention periods had the focus on improvements of endurance performance, because, in adolescence, athletes’ capacity should be stepwise developed and optimized (Myer et al., 2015). Future evaluations should assess longitudinal data including individual senior success to identify performance predictors and drop-out reasons.

It must be noted, that the presented results are partially based on self-reported data, in particular the parameters health sense and stress level. The aim was to detect abnormalities, so an impaired health sense could indicate an existing infection. In the analyzes, low health senses were associated with increased prevalences of clinical complaints, but not with higher hs-CRP values. However, in contrast to hs-CRP, the parameter health sense showed a relationship to the end point performance progress. Subjective self-reported data will not replace objective parameters, but they are able to suggest individual impaired sensations. In this regard, training can be modulated to avoid further complications. Therefore, the monitoring of subjective self-reported data, in particular because of their close association to performance development, can represent an appropriate diagnostic tool to control training. Nevertheless, the clinical relevance of such health-related parameters remains to be further investigated (e.g., validated questionnaires [WURSS], blood samples) (Barrett et al., 2005).

Despite similar training modalities, performance development varies among each athlete. The degree of progress can be influenced by numerous parameters, such as training quality and quantity, genetic disposition, health, physiological conditions, and psychosocial factors. Nearly two-thirds of all athletes demonstrated an improvement of performance, one-third of at least 7.5%. These groups reported improved health senses and lower stress levels. In contrast, objective known indicators, such as hemoglobin concentrations and vitamin D values, offered no differences between the groups. Therefore, subjective parameters, which reflect athletes’ health, should be considered to regulate training, and finally, to predict performance potential. Hence, already at a young age ensuring health should be one superior aim, resulting in a higher training quality, and thus, in a better performance development.

The analyzes based on data from a large, prospective, controlled study of adolescent athletes (Blume et al., 2018). Therefore, “real-life” results could be determined, which in turn can be transferred and applied directly in practice. Beside known, or rather assumed biochemical markers regarding performance development prediction, the evaluation of subjective parameters was focus of the analysis. Although these were determined subjectively, the assessment was performed standardized and, in addition, longitudinal. The results emphasize the consideration of such parameters for health evaluation, training monitoring and performance prediction. However, biochemical markers should not be disregarded, in particular for the detection of abnormalities, such as deficiency, anemia or a systemic inflammation. Rather, the individuals’ performance development is based on numerous influencing factors, which all should be estimated. In contrast to the benefits of a “real-life” study, it comprises limitations. In order to maintain compliance and to ensure practicability, long questionnaires and complex laboratory measurements were left unconsidered. In further analyzes these methods should be compared with the used tools for validation. The results were confirmed for various athletes of different sports. Nevertheless, a larger homogeneous collective is needed to verify the results (e.g., similar basic values, same performance test). Also, individuals’ long-term and sport-specific performance should be identified. The focus of this analysis was the development of the athletes’ endurance capacity, therefore, endurance-related sports and training periods were included. As already mentioned, training hours should not equalize with the total training load. For this purpose, further parameters should be determined (e.g., detailed training information such as frequency, intensity and duration). Finally, the evaluation of subjective parameters represents, in combination with other variables, such as known biochemical markers, a practicable tool for training monitoring und performance prediction.

## Data Availability Statement

The datasets generated for this study are available on request to the corresponding author.

## Ethics Statement

The studies involving human participants were reviewed and approved by the Medical Research Ethics Committee (TU München). Written informed consent to participate in this study was provided by the participants’ legal guardian/next of kin.

## Author Contributions

KB and BW conceived the present idea, developed the theory, performed the computations, verified the analytical methods, discussed the results, and contributed to the final manuscript.

## Conflict of Interest

The authors declare that the research was conducted in the absence of any commercial or financial relationships that could be construed as a potential conflict of interest.
